# Pharmacokinetic-Pharmacodynamic Modeling for Coptisine Challenge of Inflammation in LPS-Stimulated Rats

**DOI:** 10.1038/s41598-018-38164-4

**Published:** 2019-02-05

**Authors:** Yingfan Hu, Li Wang, Li Xiang, Jiasi Wu, Wen’ge Huang, Chensi Xu, Xianli Meng, Ping Wang

**Affiliations:** 1 College of Pharmacy, Chengdu University of Traditional Chinese Medicine, Chengdu, 611137 Sichuan China; 2 Chengdu Pharmoko Tech Corp., Ltd., Chengdu, 610041 China

## Abstract

Pro-inflammatory factors are important indicators for assessing inflammation severity and drug efficacy. Coptisine has been reported to inhibit LPS-induced TNF-α and NO production. In this study, we aim to build a pharmacokinetic-pharmacodynamic model to quantify the coptisine time course and potency of its anti-inflammatory effect in LPS-stimulated rats. The plasma and lung coptisine concentrations, plasma and lung TNF-α concentrations, plasma NO concentration, and lung iNOS expression were measured in LPS-stimulated rats after intravenous injection of three coptisine doses. The coptisine disposition kinetics were described by a two-compartment model. The coptisine distribution process from the plasma to the lung was described by first-order dynamics. The dynamics of plasma TNF-α generation and elimination followed zero-order kinetics and the Michaelis-Menten equation. A first-order kinetic model described the TNF-α diffusion process from the plasma to the lung. A precursor-pool indirect response model was used to describe the iNOS and NO generation induced by TNF-α. The inhibition rates of TNF-α production by coptisine (54.73%, 26.49%, and 13.25%) calculated from the simulation model were close to the decline rates of the plasma TNF-α AUC (57.27%, 40.33%, and 24.98%, respectively). Coptisine suppressed plasma TNF-α generation in a linear manner, resulting in a cascading reduction of iNOS and NO. The early term TNF-α response to stimulation is a key factor in the subsequent inflammatory cascade. In conclusion, this comprehensive PK-PD model provided a rational explanation for the interlocking relationship among TNF-α, iNOS and NO production triggered by LPS and a quantitative evaluation method for inhibition of TNF-α production by coptisine.

## Introduction

Inflammation is an immune response to harmful stimuli, such as trauma, bacteria, and viruses. Immune cells, including T cells and macrophages, are stimulated to excrete inflammatory cytokines, such as TNF-α, IL-6, IFN-γ and NO. These inflammatory factors have been widely used as biomarkers to evaluate the inflammation severity and effects of drugs^1–4^. Previous studies have confirmed that the dynamic profiles of these biomarkers are asynchronous. *Lin*, *Nientsung* and *Lee*, *Ru Ping et al*. examined the time course of the plasma TNF-α and NO concentrations in lipopolysaccharide (LPS)-stimulated rats (intravenous infusion, 10 mg/kg within 20 min) and found that the plasma TNF-α concentration peaked within 1 h^5^, whereas the plasma nitric oxide (NO) concentration culminated at 9 h^6^. Moreover, the inflammatory response shows spatial differences. Inflammatory cytokines can transmit inflammatory signals from the area of inflammation to other tissues, participate in upregulation of the inflammatory response and aggravate the damage. Inducible nitric oxide synthase (iNOS) mRNA expression has been reported to reach a peak in the lungs of rats at 3 hours and in the liver at 6 hours after a 10 mg/kg LPS infusion, whereas low levels are found in the spleen, heart and kidney^5^. Considering these temporal and spatial differences in the dynamic profiles of biomarkers, an integrated analysis based on physiology is a reasonable method for assessing inflammatory processes, interactions between inflammatory mediators and holistic effects of drugs.

A pharmacokinetic-pharmacodynamic (PK-PD) model is a useful tool to analyze multiple biomarkers and has been broadly used to evaluate the effects of drugs on pathological processes. *Abhijit Chakraborty et al*. established a comprehensive PK-PD model to capture the dynamics of regulation of plasma NO generation by TNF-α and IFN-γ and used it to evaluate the processes, efficacy and interaction of IL-10 and prednisolone^7^. *Siddharth Sukumaran et al*. built a mechanism-based model to describe the NO production process regulated by iNOS mRNA expression in LPS-stimulated rats and quantitatively assessed the holistic anti-inflammatory effects of methylprednisolone^8^.

**Figure 1:**
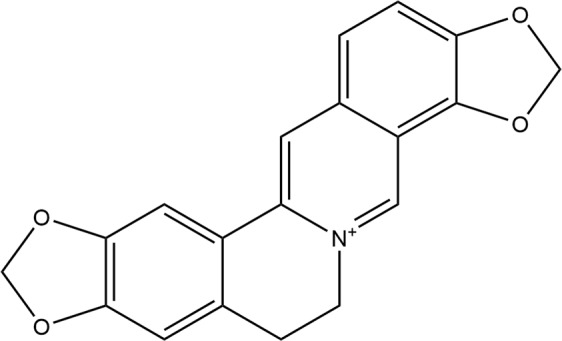
Chemical structures of the coptisine compound (source: PubChem^9^).

Coptisine, which is an isoquinoline alkaloid (Fig. 1)^9^, has many biological activities in many diseases, including inflammation, infection, and cancer^10–12^. To date, many studies have found that coptisine affects inflammatory molecule expression in several cell types^10^ and *in vivo*^13,14^. In our previous study^15^, we found that coptisine inhibited NF-κB, MAPK, and PI3K/Akt activation, suppressed iNOS expression, and decreased NO and proinflammatory cytokine (TNF-α, IL-1β, and IL-6) levels. However, these studies on the anti-inflammatory effects of coptisine were performed at selected time points and did not consider the relationships among biomarkers in the network. Therefore, these assessments may cause deviation in evaluating the therapeutic effect of coptisine. Here, a comprehensive mathematical model including the expression of multiple biomarkers during the detailed inflammatory process can be established to capture the entire inflammatory phase from excitation to response. Recently, the coptisine plasma and urine concentration-time profiles and bioavailability in rats have been reported^16–21^. However, no detailed report has investigated the relationship between drug exposure and the pharmacodynamic response to coptisine.

In this study, we established a pharmacokinetic model (PK) to describe the dynamics of coptisine. Simultaneously, we used inflammatory cytokines (TNF-α, iNOS, and NO) as evaluation indicators and built a kinetic model to describe the detailed pathological process in LPS-stimulated rats. Then, the inhibitory effect of coptisine was quantitatively added to this kinetic model to obtain the overall anti-inflammatory effect. This mechanism-based PK-PD modeling approach provided a feasible method to assess and quantify the anti-inflammatory and dose-effect relationships of coptisine through a comprehensive analysis.

## Results

### Pharmacokinetics of Coptisine

**Figure 2:**
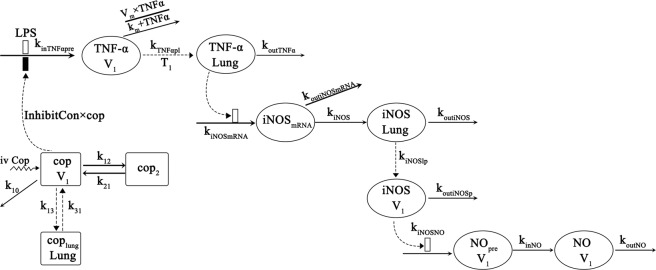
Schematic of the pharmacokinetic/pharmacodynamic model for the effects of LPS and coptisine on TNF-α, iNOS and NO generation. Coptisine is abbreviated as “cop”. “□” and “■” indicate stimulation and inhibition, respectively. The parameters are described in Table 1.

**Figure 3:**
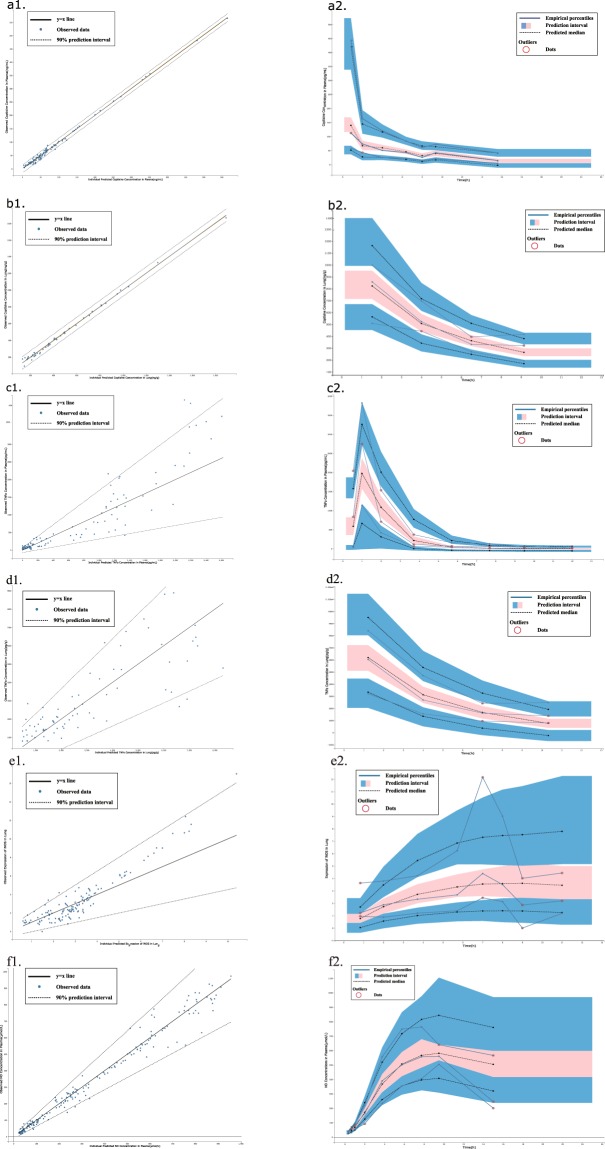
Goodness of fit plots for the final population pharmacokinetic-pharmacodynamic model. Panels a1, b1, c1, d1, e1, and f1 show the observed vs population and individual predicted data. Panels a2, b2, c2, d2, e2, and f2 show the visual predictive check (VPC) plots describing the population pharmacokinetic model. In the VPC plots, the blue solid line represents the empirical percentiles, and the shaded areas represent the 5^th^ and 95^th^ percentiles of the 2000 data sets simulated from the final model. (**a** represents the plasma coptisine concentration, **b** represents the lung coptisine concentration, **c** represents the plasma TNF-α concentration, **d** represents the lung TNF-α concentration, **e** represents iNOS expression in the lung, and **f** represents the plasma NO concentration).

A two-compartment pharmacokinetic model was built to describe the kinetics of coptisine (Fig. 2). Figure 3 displays the goodness-of-fit plots and visual predictive assessment results for the final PK model. These graphs show that the PK model fully describes individuals and population predictions for the blood and lung coptisine levels (Fig. 3a,b). The final pharmacokinetic parameter estimation results are shown in Table 1. The area under the drug concentration curve (AUC), which represented drug exposure in the plasma or lungs, was calculated under the trapezoidal rule. A linear relationship was found between the coptisine dose and the AUC_0–12h_ of the plasma-drug concentration (AUC_0–12h,7.74_ = 1173.01 ng·h/mL; AUC_0–12h,3.87_ = 492.57 ng·h/mL; and AUC_0–12h,1.94_ = 289.79 ng·h/mL). The AUC_0–12h_ values for lung coptisine were 5082.12 and 3123.74 ng·h/mL for the LPS + coptisine 7.74 mg/kg and LPS + coptisine 3.87 mg/kg groups, respectively. In these two dosing groups, the AUC ratios of lung coptisine and coptisine were 4.33 and 6.34, respectively. The lung-to-plasma AUC ratios were greater than one, which meant that coptisine was readily distributed to the lung. Following intravenous administration, the three coptisine doses were rapidly eliminated. The coptisine concentration in the blood declined with a t_1/2_ of 1.51 hours, which was close to that reported in previous research^22^.

**Table 1:** PK-PD Model Parameter Values.

Parameter	Definition	Estimate	CV%
*V*_1_ *(L/kg)*	Central volume of distribution	2.63×10^3^	10.29
*k*_10_*(h*^*−1*^)	Systemic clearance of coptisine	4.58×10^−1^	9.99
*k*_12_*(h*^*−1*^)	Distribution from plasma to other organizations	2.05	13.39
*K*_21_*(h*^*−1*^)	Distribution from other organizations to plasma	6.68×10^−1^	9.51
*k*_13_*(h*^*−1*^)	Distribution from plasma to lung	1.21×10^−2^	16.92
*k*_31_*(h*^*−1*^)	Distribution from lung to plasma	3.77	17.16
*k*_*0*,*0-0*.*33h*_*(pg/(mL·h*))	Formation rate of TNF-α in blood (0–0.33 h)	645.73	29.18
*k*_*0*,*0*.*33–1h*_*(pg/(mL·h*))	Formation rate of TNF-α in blood (0.33–0.67 h)	5881.32	31.11
*InhibitCon(mL/ng*)	Linear inhibitory constant of coptisine on plasma TNF-α production rate	8.83×10^−4^	47.33
*V*_*m*_*(h*^*−1*^)	Maximal saturable metabolism rate of TNF-α	5.99×10^3^	55.85
*k*_*m*_*(pg/mL*)	Concentration of TNF-α when the rate of non-linear elimination was at half its maximum value	5.18×10^3^	61.92
*k*_*TNFαpl*_*(h*^*−1*^)	Distribution from plasma to lung	3.54×10^−7^	177.12
*k*_*outTNFα*_*(h*^*−1*^)	Elimination constant of TNF-α	2.34×10^−1^	7.09
*k*_*iNOSmRNA*_*(h*^*−1*^)	Production of iNOS mRNA induced by TNF-α	3.38×10^−3^	10.85
*k*_*outiNOSmRNA*_*(h*^*−1*^)	Elimination constant of iNOS mRNA	2.36	7.75
*k*_*iNOS*_*(h*^*−1*^)	Production of iNOS from iNOS mRNA	2.99×10^−1^	10.33
*k*_*outiNOS*_ *(h* ^*−1*^ *)*	Elimination constant of iNOS	3.72	10.06
*k*_*iNOSlp*_ *(h* ^*−1*^ *)*	Distribution from lung to plasma	23.41	19.96
*k*_*outiNOSp*_ *(h* ^*−1*^ *)*	Elimination constant of iNOS in plasma	1.92	6.25
*k*_*iNOSNO*_ *(h* ^*−1*^ *)*	Production of pre-NO from iNOS	772.17	21.58
Δ	Amplification factor	1.23	29.71
*k*_*inNO*_ *(h* ^*−1*^ *)*	Production of NO from pre-NO	354.68	63.97
*k*_*outNO*_ *(h* ^*−1*^ *)*	Elimination constant of NOS	3.46	11.13

### Pharmacodynamics and PK-PD

**Figure 4:**
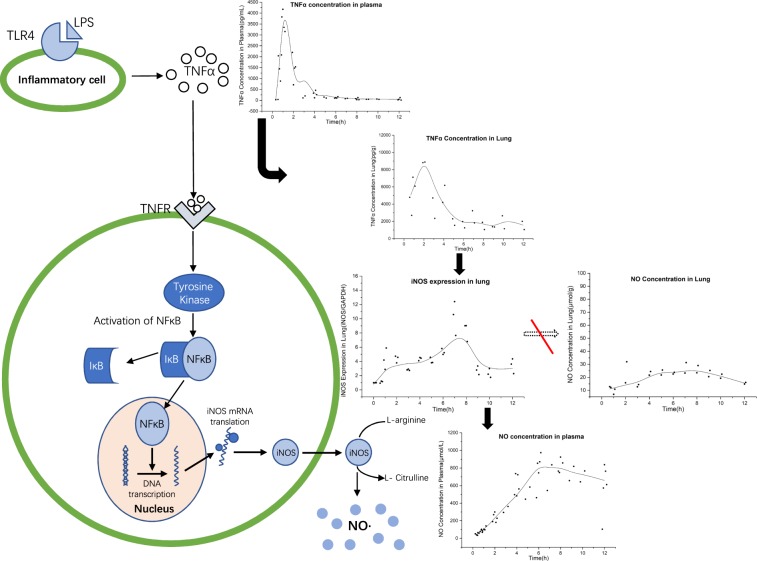
Conceptual representation of the process of TNF-α, iNOS and NO in LPS-stimulated rats. Plasma LPS binds to TLR4, causing receptor activation and TNF-α release. TNF-α in the blood is transferred to the lung, where it docks with its receptor (TNFR) and activates NF-κB. Activated NF-κB enters the cell nucleus and binds to the iNOS gene promotor region to activate transcription and iNOS generation. Then, lung iNOS diffuses into the blood and results in NO overexpression. The time curve graph is described as a scatter plot with its locally weighted scatterplot smoothing (LOWESS) line. The jitter procedure was used to show complete individual data.

The schematic of the integrated PK-PD model is presented in Fig. 2. We provided a schematic to show the process after LPS stimulation in rats using a circle and arrow (Fig. 4). When LPS enters the blood, TNF-α was elevated by approximately 2000 times at 1 h post-LPS-dosing and then rapidly declined back to the baseline value. A zero-order increase formula was used to describe the TNF-α elevation, which showed two stages with different generation rates (Table 1, Fig. S1). The lungs of the rats lacked pulmonary intravascular macrophages (PIMs), which resulted in insensitivity to the low LPS concentration. Additionally, intravenous injection of a low LPS dose (100 μg/kg) did not increase the lung LPS concentration (Fig. S2). Based on the above two points, we speculated that TNF-α in the lungs diffused from the blood during the early stages of acute inflammation. Therefore, we used a diffusion model to describe the delayed kinetics of the lung TNF-α concentration (peaked at 2 h). We observed a further delay in lung iNOS expression. Approximately 7 hours was required from the initiation of inflammation to iNOS expression, which agreed with our *in vitro* result. The *in vitro* study showed that the peak of iNOS expression appeared at 8 h in LPS-stimulated RAW264.7 cells (Fig. S3). Since no variation was observed in the lung NO concentration (Fig. S4), we could infer that the lung iNOS was transferred to the plasma and induced NO production. The serum NO peak was captured at 8 h (approximately 20-fold). A similar model of iNOS-mediated NO production was reported by *Siddharth Sukumaran et al*.^8^.

This dynamic model used TNF-α as a key factor in the inflammatory cascade and provided a quantitative description of the change patterns of TNF-α, iNOS and NO in the plasma and lung. The time-delay cascade processes and peak times predicted by this model fitted with the observed data.

Coptisine inhibited TNF-α generation in the blood in a linear manner, and its inhibitory constant was calculated as 8.83 × 10^−4^ mL/ng. According to the calculation from the simulation model, the maximum plasma concentration (C_max_) of the three coptisine doses inhibited plasma TNF-α production by approximately 54.73%, 26.49%, and 13.25%. The rate of decline calculated from the mathematical model was close to the descent rate of the plasma TNF-α AUC. The AUC_0-12h_ values of plasma TNF-α were 5461.63, 2333.54, 3258.53, and 4097.53 pg·h/mL for the LPS, LPS + coptisine 7.74 mg/kg, LPS + coptisine 3.87 mg/kg, and LPS + 1.94 mg/kg groups, respectively. Compared with that of the LPS group, the ratios of the plasma TNF-α AUC decreased by 57.27%, 40.33%, and 24.98%, respectively. Thus, it could be confirmed that coptisine inhibited plasma TNF-α production in a linear manner.

**Figure 5:**
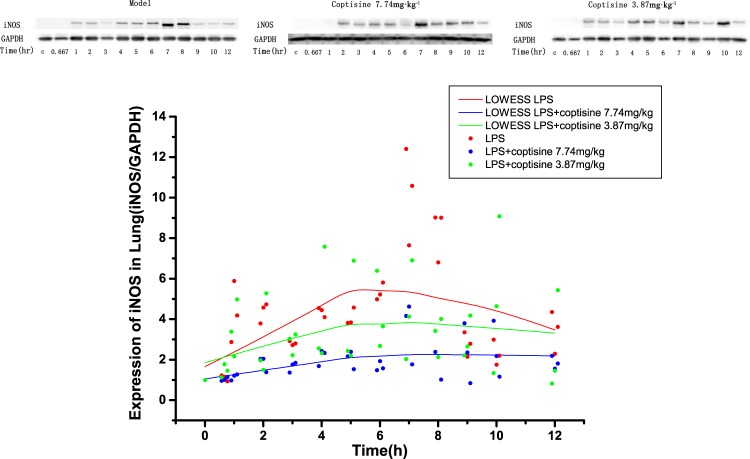
Western blotting analysis of lung iNOS in rats stimulated with LPS (100 μg/kg) following administration of coptisine at doses of 7.74 mg/kg and 3.87 mg/kg. After drug administration, the lungs were surgically removed at the indicated time points, immediately snap-frozen in liquid nitrogen, and stored at −80 °C prior to analysis. The lung lysate was prepared in RIPA containing PMSF and further resolved with 10% SDS-PAGE gels. iNOS was detected with a rabbit monoclonal anti-iNOS antibody (1:1000 dilution). iNOS and GAPDH shown in the same group are from the same gel. The bands have been cropped. The full-length blots are presented in Fig. S7. The results were described as a scatter plot with its locally weighted scatterplot smoothing (LOWESS) line. The colors assignment for the groups are red, LPS; blur, LPS + coptisine 7.74 mg/kg; green, LPS+ coptisine 3.87 mg/kg. The jitter procedure was used to show complete individual data.

The reduction of inflammatory cytokines in the later phase (iNOS and NO) should present linear dependence on the coptisine dose because the model is a tandem model. The Western blotting results for iNOS expression are shown in Fig. 5, which presents the protein levels at various time points after LPS stimulation. The AUC_0–12h_ values of lung iNOS were 55.75, 28.2, and 30.72 (LPS, LPS + coptisine 7.74 mg/kg and LPS + coptisine 3.87 mg/kg, respectively). Compared with that of the LPS group, the ratios were decreased by 49.43% and 44.9%, respectively. The AUC_0–12h_ plasma NO values for the four groups were 6447.87, 3924.59, 4072.68, and 7381.51 μmol·h/L (LPS, LPS + coptisine 7.74 mg/kg, LPS + coptisine 3.87 mg/kg, and LPS + 1.94 mg/kg, respectively). Compared to that of the LPS group, the ratios were reduced by 39.13%, 36.84% and a negative value, respectively. The AUCs of TNF-α, iNOS and NO showed the same trends with the coptisine doses, which proved that inhibition of TNF-α by coptisine could cause a cascading decrease in NO.

The visual prediction and evaluation of the final PK-PD model are presented in Fig. 3. For the plasma and lung TNF-α concentrations, the PK-PD model properly estimated the observed data, and the relationship between the observations and population predictions was well correlated. Furthermore, these graphs indicated that the model appropriately described the individual and population predictions of the pharmacodynamic indicators and that these pharmacodynamic model parameters were well estimated. The values of Akaike and Bayesian information criteria are −7655.67 (AIC) and −7488.59 (BIC), respectively. This comprehensive and complex PK-PD model based on physiology correctly captured the intracorporal processes, drug efficacy, and dose-response relationships. However, the fitting result of iNOS fluctuated more than that of the other indicators due to individual differences among the rats.

## Discussion

Endotoxin can cause uncontrolled activation of immune responses to generate inflammatory cytokines^23^. Pro-inflammatory factors, such as TNF-α, upregulate the inflammatory response and cause tissue damage, even in the absence of infection. As biomarkers, generally inflammatory mediators are used to evaluate disease pathological processes and drug properties. The time courses of different biomarkers vary. Researchers found that the rat plasma TNF-α concentration peaked 1 h after endotoxin treatment, whereas the plasma NO concentration peaked at 9 h^5,6,24^. *Ayse ER et al*. found that the IL-1β concentration in the bronchoalveolar lavage fluid of rats after LPS-induced lung inflammation peaked at 1 h, whereas the peak IL-6 level occurred at 4 h^25^. Moreover, the inflammatory cytokine levels in various tissues are different. Inflammatory factors act as agents to transfer inflammatory signals from infected areas to other tissues and activate immune responses in uninfected areas. The experiment demonstrated that iNOS mRNA expression was different in various rat tissues after LPS infusion at a 10 mg/kg dose^5^. The peak of iNOS mRNA expression in the lung was detected at 3 h and the highest iNOS mRNA expression in the liver was found at 6 h, but iNOS mRNA was expressed at low levels in the spleen, heart and kidney. These findings indicate temporal and spatial variation and complex signal transfer for biomarkers.

In fact, the proinflammatory cytokine TNF-α participates in signal transmission for endotoxin-induced NO generation and plays an important role in upregulating NO synthesis. Anti-TNF-α antibody administration had been reported to reduce LPS-induced NO release in mouse macrophages, which indicates that TNF-α is a significant mediator of NO synthesis^26,27^. *In vivo* experiments also found that treatment with both anti-TNF-α and anti-IFN-γ antibodies almost completely inhibited the plasma NO production induced by LPS^28^. Furthermore, *Abhijit Chakraborty et al*. quantitatively evaluated the TNF-α- and IFN-γ-mediated NO production processes after LPS stimulation and found that TNF-α played a major role^7^. Thus, TNF-α served as the main mediator of iNOS and NO synthesis after LPS treatment. When LPS enters the vein, it is bound by macrophages in the blood, which rapidly produces large amounts of TNF-α. The quickly generated TNF-α binds to specific receptors and activates a secondary inflammatory reaction similar to ERK, JNK and MAPK pathway activation by LPS, leading to overexpression of iNOS and NO^29–31^.

Many studies have reported that coptisine has anti-inflammatory activity both *in vitro* and *in vivo*. Coptisine has an inhibitory effect on TLR4-mediated inflammatory signaling pathways. Our previous research confirmed that coptisine could prevent IκBα degradation and ERK, JNK, MAPK and PI3k/Akt phosphorylation in LPS-stimulated mice and macrophages. Coptisine effectively inhibited the iNOS, IL-1β and IL-6 mRNA levels and iNOS, NO and cytokine production. In the supplementary materials (Fig. S5), coptisine was added into medium after 0.5 h or 4 h LPS stimulation, we compared the NO production secreted by RAW264.7 cells and found that coptisine (30 μM) could decrease the NO concentration after LPS administration 0.5 h, while the inhibition was weaker after LPS stimulation 4 h. The addition of coptisine during the rapid TNF-α generation stage at 0.5 h after LPS administration reduced the NO level. In comparison, when TNF-α was produced in large amounts at 4 h after LPS administration, coptisine had little inhibitory effect on NO. This result suggested that coptisine reduced the iNOS and NO levels by inhibiting TNF-α.

Considering the correlation among biomarkers, we established a mechanism-based PK-PD model to integrate TNF-α, iNOS and NO and to clarify the intrinsic connections among the biological and anti-inflammatory mechanisms of coptisine. Our results demonstrated that coptisine linearly suppressed the blood TNF-α production induced by LPS in a dose-dependent manner, resulting in cascade diminution of iNOS and NO expression. The plasma TNF-α AUC_0–12h_ values were decreased by approximately 24.98% to 57.27% by the three coptisine doses in a dose-dependent manner. Compared with that of steroids and cytokines, prednisolone (IC_50_ = 171 ng/mL) efficiently suppressed LPS-induced TNF-α, and IL-10 (IC_50_ = 0.057 ng/mL) was also effective at controlling TNF-α release. The inhibitory rates of the TNF-α AUC_0–6h_ were 82% (prednisolone) and 93% (IL-10)^7^. To increase the rate of TNF-α inhibition, the maximum blood concentration of coptisine should be approximately 1200 ng/mL, and the intravenous dose of coptisine should be increased to 15 mg/kg. The median lethal dosage (LD_50_) of berberine through intravenous injection is 9.0386 mg/kg^32^. Coptisine has the same typical natural benzyl tetrahydroisoquinoline alkaloid skeleton as berberine. Thus, the LD_50_ of coptisine may be close to that of berberine. The maximum effective dose of coptisine is much higher than the LD_50_. In addition, the low content in herbs and the oral absolute bioavailability (<2%)^33^ cause a poor overall anti-inflammatory efficacy for coptisine. These results suggest that the efficacy of coptisine may need to be improved through pharmaceutics or structural modification.

In conclusion, early generation of TNF-α is a key factor in the subsequent inflammatory cascade. Coptisine decreased plasma TNF-α production in a linear manner, followed by a reduction in the iNOS and NO levels in this rat inflammation model. This integrated mechanism-based PK-PD model described the individual TNF-α and NO concentrations and iNOS expression and reasonably explained the lag and relationships among these inflammatory factors. This approach not only successfully modeled the experimental data but also provided a reasonable method to quantitatively clarify the process and target by which coptisine inhibits the inflammatory response. We believe that PK/PD studies may be a useful approach to explain the active mechanism and therapeutic response of coptisine and to screen experimental designs for preclinical and clinical research.

## Materials and Methods

### Chemicals and reagents

Coptisine (standard powder, 99% pure) and berberine chloride (Internal Standard, IS) were obtained from Must Biotechnology, Inc. (Chengdu, China). Lipopolysaccharide (Escherichia coli 055:B5) was purchased from Sigma-Aldrich Co., LLC. (St. Louis, MO, USA). Methanol and acetonitrile (HPLC-grade) were obtained from Thermo Fisher Scientific Inc. (Fair Lawn, NJ, USA). The BCA protein assay kit was obtained from Thermo Fisher Scientific (Waltham, MA, USA). The antibody to iNOS (Cat. NO. ab178945) was purchased from Abcam Company Ltd. (Cambridge, MA, USA). The antibody to GAPDH (Cat. NO. 200306-7E4) was obtained from Zen Bioscience (Chengdu, China). The coptisine injection was prepared in accordance with a previous study^15^.

### Animals

Male Sprague–Dawley (SD) rats (200 ± 20 g) were obtained from the Institute of Laboratory Animals of Sichuan Academy of Medical Sciences & Sichuan Provincial People’s Hospital (Chengdu, Sichuan). The rats were acclimatized in a room with a stationary temperature (22 ± 2 °C) and light: dark cycle (12 h:12 h) for at least 1 week before the experimental procedures. The experiments were conducted under the experimental practices and standards approved by the Animal Welfare and Research Ethics Committee at Chengdu University of TCM. All experiments were performed in accordance with the National Institute of Health Guide for the Care and Use of Laboratory Animals. This study was approved by the Animal Experiment Committee of Chengdu University of TCM. We made every effort to minimize pain.

### Experimental Design

Table 2 generalizes the experimental design of this study.

**Table 2:** Overview of the Experimental Design.

Groups	Number of animals	LPS dose (0 h, μg/kg)	Coptisine dose (0.5 h, mg/kg)	Types of measurements and time points for collection of these measurements
***Study 1***				
1A	3	100	—	Collection of blood via the tail vein at each time point: 0.667, 1, 2, 4, 6, 8, 12 and 24 h. Measurement of coptisine concentration.
1B	3	100	7.74	
1C	3	100	3.87	
***Study 2***				
2A	3	100	—	Collection of blood via the tail vein at each time point: 0, 0.333, 0.667, 1, 2, 4, 6, 8, 12 and 24 h. Measurement of plasma TNF-α and NO concentrations.
2B	3	100	7.74	
2C	3	100	3.87	
2D	3	100	1.94	
***Study 3***				
3A	2	—	—	Rats were anesthetized with urethane. Collection of blood and lung tissues at each time point: 0 (group 3A), 0.667, 1, 2, 3, 4, 5, 6, 7, 8, 9, 10 and 12 h. (groups 3B, 3C, and 3D, n = 2 per group per time point). Measurement of coptisine concentration in the plasma and lung, TNF-α and NO concentrations in the plasma and lung, and iNOS expression in the lung.
3B	24	100	—	
3C	24	100	7.74	
3D	24	100	3.87	
***Study 4***				
4A	3	—	—	Rats were anesthetized with urethane. Collection of blood and lung tissues at each time point: 0 (group 4A), 2, 4, 6, 7, 8 and 9 h. (group 4B, 4C, and 4D, n=3 per group per time point). The detection was the same as *Study 3*.
4B	18	100	—	
4C	18	100	7.74	
4D	18	100	3.87	

#### Study 1: Pharmacokinetics Study of Coptisine in LPS-Stimulated Rats

Three rats were randomly divided into each of three groups (1A, 1B and 1C). All of the rats received a 100 μg/kg LPS injection (dissolved in sterile saline) via the tail vein (time = 0). The animals in groups 1B and 1C received coptisine (Cop, 7.74 mg/kg and 3.87 mg/kg, respectively) by intravenous injection in the tail after 0.5 h. Blood samples were collected with tubes (AXYGEN Micro Tube, Corning Incorporated, NY, USA) soaked with heparin sodium via the tail vein at the 0.5, 1, 2, 4, 6, 8, 12 and 24 h time points.

All samples were centrifuged for 15 min at 3500 rpm and 4 °C. Plasma was preserved at eighty degrees below zero centigrade until analysis. At the time of analysis, plasma (50 μL) was mixed with acetonitrile (200 μL) to precipitate proteins. After high-speed centrifugation (13000 r/min, 10 min), the clear upper fluid was collected and evaporated with a flow N_2_ gas, re-dissolved in 100 μL of acetonitrile/0.1% formic acid (40/60 v/v), and detected using a liquid chromatography (LC) system. Standard solutions and quality control were both taken into account. Berberine was used as an internal standard.

The LC system was an Ultra-Fast Liquid Chromatography System (SHIMADZU Nexera UFLC LC-30A) with a Zorbox RRHD Eclipse Plus-C8 (1.8 μm, 3.0 × 150 mm) column (Agilent Technologies, Santa Clara, CA, USA). The mobile phase consisted of an ACN (A) and 0.1% formic acid (B) gradient elution as follows: 90%~60% B (0~10 min), 60% B (10~20 min), and 90% B (20~25 min). The mobile phase was delivered at a constant flow rate of 0.7 mL/min. The detection wavelength was 345 nm, the injection volume was 50 μL, and the oven temperature was maintained at 40 °C.

#### Study 2: Pharmacodynamics Study of Coptisine in LPS-Simulated Rats

SD rats (n = 12) were divided randomly into 4 groups (2 A, 2B, 2 C, and 2D). Each group received 100 μg/kg of LPS via the tail vein (time = 0). After 0.5 h (time = 0.5 h), the rats in groups 2B, 2 C and 2D were injected with coptisine (Cop, 7.74 mg/kg, 3.87 mg/kg, and 1.94 mg/kg, respectively). Sampling through the tail vein was carried out at the 0, 0.333, 0.667, 1, 2, 4, 6, 8, 12 and 24 h time points. The blood TNF-α concentration was measured using an ELISA kit (Multisciences Co., Ltd. Hangzhou, Zhejiang) following the manufacturer’s instructions. The NO concentration was determined using nitrate reductase acquired from the Nanjing Jiancheng Bioengineering Institute (Nitric Oxide Assay Kit, Nanjing, Jiangsu).

#### Study 3: Pharmacokinetics-Pharmacodynamics Study of Coptisine in LPS-Stimulated Rats

Animals (n = 74) were divided randomly into 4 groups (3A, 3B, 3C, and 3D). The rats in groups 3B, 3C, and 3D were injected with 100 μg/kg of LPS via the tail vein (time = 0) and then received vehicle (rats in group 3B) or coptisine (the rats in groups 3C and 3D received 7.74 mg/kg and 3.87 mg/kg, respectively) by intravenous injection in the tail after 0.5 h. Blood was sampled from the abdominal artery of rats anesthetized with urethane, and the lungs were collected immediately, frozen in liquid nitrogen and kept at minus eighty degrees centigrade. The corpses were disposed of properly. For animals in the control group (group 3A), samples were collected and denoted as 0 h. In groups 3B, 3C and 3D, blood was sampled at the 0.667, 1, 2, 3, 4, 5, 6, 7, 8, 9, 10 and 12 h time points (n = 2 per group per time point).

The lung tissues were homogenized with RIPA (radioimmunoprecipitation assay buffer) including PMSF (phenylmethylsulfonyl fluoride) using a tissue homogenizer. After high-speed centrifugation, the treatment of the homogenate supernatants was similar to that of the blood samples. The coptisine concentrations in the lungs and plasma were detected by the LC system. Berberine was included as an internal standard. The mobile phase consisted of an ACN (A) and 0.1% formic acid (B) gradient elution as follows: 90~60% B (0~10 min), 60% B (10~20 min), and 90% B (20~25 min). The mobile phase was delivered at a constant flow rate of 0.7 mL/min. The detection wavelength was 345 nm, the injection volume was 50 μL, and the oven temperature was maintained at 40 °C. The TNF-α and NO concentrations in the lungs were quantified as described in *Study 2*.

Pharmacodynamic research was established for iNOS expression in the lung as determined by western blotting. The total protein concentration in the lung was determined with the BCA protein assay kit. Samples with equal amounts of protein were separated using a 10% SDS polyacrylamide gel and then transferred to a polyvinylidene fluoride membrane (Millipore, Bedford, MA, USA). The membrane was blocked with 5% BSA (bovine serum albumin) dissolved in TBST (Tris-buffered saline with Tween 20). The membrane was incubated with a specific antibody (1:2000 dilution) overnight (4 °C) and then with TBST containing an HRP-conjugated secondary antibody (ZenBioScience) for 1 h at room temperature. The bands were scanned with the ChampChemi 610 Plus Imager system (Beijing Sage Creation Science Co., Ltd.). The grayscale value of each band was detected by a computer image analysis system (Quantity One, Bio-Rad). GAPDH, which is considered a housekeeping protein, was measured simultaneously to standardize the iNOS level. The iNOS expression level at each time point were normalized to the control iNOS level (*iNOS*_0_, group 3A, time = 0 h) and calculated as follows^34^:

$${\rm{Relative}}\,{\rm{iNOS}}\,{\rm{ratio}}=\frac{iNOS/GAPDH}{iNO{S}_{0}/GAPD{H}_{0}}$$

#### Study 4: Pharmacokinetics-Pharmacodynamics Study of Coptisine in LPS-Simulated Rat

Animals (n = 57) were divided randomly into 4 groups (4A, 4B, 4C, and 4D). The rats in each group received the same treatment as described in *Study 3*. Blood was sampled at the 0 (group 4A), 2, 4, 6, 7, 8, and 9 h time points (n=3 per group per time point), and the lungs were collected at the same time. The plasma and lung coptisine concentrations, plasma TNF-α and NO concentrations, and lung iNOS expression levels were measured.

### PK-PD Modeling

Fig. 2 shows a schematic of the integrated PK-PD model. The model describes the dynamic changes in the TNF-α, iNOS and NO concentrations in the plasma and lung, the disposition of coptisine, and the inhibition of coptisine during this process. In this section, the compartment models (one or two), absorption (zero or first order), lag time (with or without), and elimination process (linear or nonlinear) were explored. The best model was selected according to Akaike’s information criterion (AIC), goodness-of-fit (GOF) plots, and the coefficient of variation (CV). The data in this paper consist of 4 studies that sacrificed 152 rats and the corresponding 1118 data points, which were used for modeling simultaneously.

Considering that the PK and PD data were all obtained from LPS-stimulated male rats with fewer biometric differences, we did not introduce covariates, such as weight or sex, into the PK or PD model. The estimation of the final parameters was conducted using mixed effects methods under no covariate, as described in detail below.

#### PK Model

The plasma coptisine concentration was evaluated after administration of the 1.94, 3.87 and 7.74 mg/kg doses. We developed a two-compartment pharmacokinetic model containing linear elimination.

1 $$\frac{d{\rm{cop}}}{dt}=-\,{k}_{10}\times cop-{k}_{12}\times cop+{k}_{21}\times co{p}_{2}$$

2 $$\frac{dco{p}_{2}}{dt}={k}_{12}\times cop-{k}_{21}\times co{p}_{2}$$

The plasma coptisine concentration is *cop*, *cop*_2_ is the peripheral compartment, *k*_10_ represents systemic clearance, and *k*_12_ and *k*_21_ are distribution rate constants between the plasma and peripheral compartment.

Coptisine in the lung was distributed from the plasma, which we described using the following formula:

3 $$\frac{dco{p}_{lung}}{dt}={k}_{13}\times cop-{k}_{31}\times co{p}_{lung}$$

where *cop*_*lung*_ represents the coptisine concentration in the lung, and *k*_13_ and *k*_31_ represent the distribution of coptisine between the plasma and lung, respectively.

#### PD Model

Fig. 4 shows the process of TNF-α, iNOS and NO in rats after LPS stimulation. Leukocytes in the blood were activated, followed by TNF-α production once endotoxin entered the vein. Since the plasma TNF-α of rats in the model group was rapidly generated within 1 h after LPS stimulation, we used two zero-order kinetic models to describe plasma TNF-α production; the same models were proposed previously^3,4,7^. Moreover, because the generation process is presented in two stages (Fig. S1), we used a piecewise function to characterize the formation rate of TNF-α (*k*_0_) in the blood. A similar model was reported by *Abhijit Chakraborty et al*.^7^. Because of the high elimination rate of TNF-α after 2 hours of LPS stimulation, the model with Michaelis–Menten elimination was considered to quantify the serum TNF-α concentration after comparison with the zero- and first-order eliminations. Thus, we described the dynamics of plasma TNF-α generation and elimination as follows:

4 $$\frac{dTNF\alpha }{dt}={k}_{0}-\frac{{V}_{m}\times TNF\alpha }{{k}_{m}+TNF\alpha }$$

where *k*_0_ represents the zero-order production rate constant of plasma TNF-α, which has different values at the two different time periods (Table 1). *V*_*m*_ indicates the maximal rate of saturable metabolism, and *k*_*m*_ represents the TNF-α concentration when the rate of non-linear elimination is at half its maximum value. *TNFα* indicates the plasma TNF-α concentration.

Low endotoxin concentrations can barely induce pulmonary inflammation. Once endotoxin enters the vein, macrophages in the blood directly take up and bind the endotoxin. In addition, rodents lack pulmonary intravascular macrophages (PIMs), which causes their lungs to be insensitive to endotoxin^35^. Rats have been reported to require a mg/kg LPS dose to induce lung injury^36^, and pulmonary inflammation does not occur with μg/kg doses of LPS stimulation^37^. Moreover, the IKK and NFκB responses to LPS presented a dose-dependent relationship and were not obvious at the 1 ng/mL LPS dose in a single-cell system^38^. Our supplementary materials found no significant changes in the lung LPS concentrations after LPS (100 μg/kg) injection via the rat tail vein (Fig. S2). The total amount of TNF-α in the lung was much lower than that in the blood (Fig. S6). Thus, we deduced that the TNF-α in the lung was translocated from the blood rather than mediated by endotoxin in the lung. The delay in the lung TNF-α concentration was explained with a simple diffusion model.

5 $$\frac{dTNF{\alpha }_{lung}}{dt}={k}_{TNF\alpha pl}\times TNF\alpha -{k}_{outTNF\alpha }\times TNF{\alpha }_{lung}$$

where *TNFα*_*lung*_ represents the TNF-α concentration in the lung, *k*_*TNFαpl*_ is the first-order transfer rate from the plasma to the lung, and *k*_*outTNFα*_ is the elimination constant of lung TNF-α.

iNOS and sequential NO production have been proven to be mediated by TNF-α rather than directly induced by LPS. *In vitro* results have demonstrated that an anti-TNF-a antibody can inhibit nitrite production in LPS-stimulated mouse macrophage cells^26,27^. Another *in vivo* study has clarified that NO production after LPS stimulation is mediated by both endogenous TNF-α and IFN-γ^28^. Mice treated with TNF-α and IFN-γ blockers together nearly avoid plasma NO production 6 hours after LPS injection. When pretreated with anti-TNF-α or anti-IFN-γ alone, a certain amount of NO production is still detected in the blood after endotoxin stimulation. Furthermore, *Abhijit Chakraborty et al*.^7^ established a model to quantify the processes by which TNF-α and IFN-γ induced NO production after LPS challenge and found that the rate constant of TNF-α-induced NO production following LPS stimulation was much larger than that of IFN-γ-induced NO production (2155 h^−1^ vs 0.13 h^−1^, which was nearly 20,000 times higher). Accordingly, we took TNF-α as the most important mediator of iNOS and NO production in an inflammatory dynamic model induced by LPS. We depicted the effect of TNF-α in the lung on iNOS overexpression as follows:

6 $$\frac{diNO{S}_{mRNA}}{dt}={k}_{iNOSmRNA}\times TNF{\alpha }_{lung}-{k}_{outiNOSmRNA}\times iNO{S}_{mRNA}$$

7 $$\frac{diNOS}{dt}={k}_{iNOS}\times iNO{S}_{mRNA}-{k}_{outiNOS}\times iNOS$$

*k*_*iNOSmRNA*_, *k*_*outiNOSmRNA*_, and *k*_*iNOS*_ are the intercompartmental rate constants, which describe iNOS production from TNF-α. *iNOS* and *iNOS*_*mRNA*_ represent the iNOS and iNOS mRNA levels in the lung. *k*_*outiNOS*_ is the first-order iNOS elimination rate. The western blotting analysis for iNOS in the lungs is presented in Fig. 5.

In consideration of the lack of fluctuation in NO production in the lung (Fig. S4) and the peak in the serum concentration at 8 h after endotoxin injection, we inferred that iNOS in the lung diffused into the blood. We used an indirect response model with mono-compartment generation and linear system elimination to explain the dynamic changes in the serum iNOS levels.

8 $$\frac{diNO{S}_{p}}{dt}={k}_{iNOSlp}\times iNOS-{k}_{outiNOSp}\times iNO{S}_{p}$$

where *iNOS*_*p*_ represents the iNOS in the plasma, *k*_*iNOSlp*_ is the first-order transfer rate from the lung to the plasma, and *k*_*outiNOSp*_ is the elimination constant of iNOS in the plasma.

As soon as iNOS diffuses into the blood from the lung, NO is generated from L-arginine. The NO formation process was described with following formula.

9 $$\frac{dN{O}_{pre}}{dt}={k}_{iNOSNO}\times iNO{S}_{p}^{{\rm{\Delta }}}-{k}_{inNO}\times N{O}_{pre}$$

10 $$\frac{dN{O}_{plasma}}{dt}={k}_{inNO}\times N{O}_{pre}-{k}_{outNO}\times N{O}_{plasma}$$

*k*_*iNOSNO*_ indicates the NO produced by iNOS. *NO*_*plasma*_ represents the amount of nitrite (NO_2_^−^) and nitrate (NO_3_^−^), and k_*outNO*_ is the systemic clearance of NO. A similar model was adopted by *Sukumaran et al*.^8^.

#### PK-PD Model

Based on these findings, we established a direct reaction model to describe the effect of coptisine.

11 $$\frac{dTNF\alpha }{dt}={k}_{0}\times (1-f(cop))-\frac{{V}_{m}\times TNF\alpha }{{k}_{m}+TNF\alpha }$$

12 $$f(cop)=InhibitCon\times cop$$

After comparison with several models, such as the log-linear, E_max_, and sigmoid E_max_ models, we used a linear model marked as *f*(*cop*) to describe the inhibitory effect of coptisine on TNF-α formation. *cop* is the serum coptisine concentration. “*InhibitCon*” represents the linear inhibitory constant of the plasma coptisine concentration for the plasma TNF-α production rate.

#### Data Analysis and Model Qualification

The PK-PD analysis was performed with the non-linear mixed effects (NLME) modeling program in the Monolix® 2016R1 software (Antony, France: Lixoft SAS, 2016. http://lixoft.com/products/monolix/). All model parameters were fitted simultaneously, and each of the between-animal variability (BAV) values was assumed to be log-normally distributed. The residual variability, which is the level of variance between the observations and their subject-specific predictions, is described with an additive model following a normal distribution with a zero mean and ${{\rm{\sigma }}}_{add}^{2}$ variance.

To validate the reliability of the final model, the diagnostic predictive performance was assessed by normalized prediction distribution errors (NPDEs), visual predictive checks (VPCs) and a numerical predictive check (NPC) from 2000 model simulations.

## Supplementary information

Supplementary Materials for Pharmacokinetic-Pharmacodynamic Modeling for Coptisine Challenge of Inflammation in LPS-Stimulated Rats
