# Supplementary material for: Pharmacokinetic-Pharmacodynamic Modeling for Coptisine Challenge of Inflammation in LPS-Stimulated Rats
Source: Sci Rep. 2019 Feb 5;9:1450. doi: 10.1038/s41598-018-38164-4 (PMC6363730; doi:10.1038/s41598-018-38164-4)
Supplement: Supplementary file 1 — Supplementary Materials for Pharmacokinetic-Pharmacodynamic Modeling for Coptisine Challenge of Inflammation in LPS-Stimulated Rats [file 41598_2018_38164_MOESM1_ESM.docx]

**Supplementary Materials for**

**Pharmacokinetic-Pharmacodynamic Modeling for Coptisine**

**Challenge of Inflammation in LPS-Stimulated Rats**

Yingfan Hu^1^*, Li Wang^1^*, Li Xiang^1^, Jiasi Wu^1^, Wen’ge Huang^1^, Chensi Xu^2^, Xianli Meng^1^, Ping Wang^1^

^1^*College of Pharmacy, Chengdu University of Traditional Chinese Medicine, Chengdu 611137, Sichuan, China*

^2^ *Chengdu Pharmoko Tech LTD corp, Chengdu 610041, China*

*Correspondence: Ping Wang, College of Pharmacy**, Chengdu University of Traditional Chinese Medicine,* *Chengdu 611137, Sichuan, China. E-mail:* [viviansector@aliyun.com](mailto:viviansector@aliyun.com)

**These authors contributed equally to this work.*

This PDF file includes:

Figure S1. Variation in the plasma TNF-α (ΔTNF-α) concentration after LPS injection.

Figure S2. The lung LPS concentration after LPS administration via the tail vein.

Figure S3. Time course of iNOS expression after LPS stimulation in RAW264.7 cells.

Figure S4. Time course of the lung NO concentration at various time points after LPS administration in rats.

Figure S5. Effect of coptisine on the NO concentration after 0.5 and 4 h of LPS stimulation.

Figure S6. Total amount of the TNF-α in the plasma and lung in LPS-stimulated rats.

Figure S7. Full-length iNOS (130 kDa) and GADPH (36 kDa) bands.

Figure S1. Variation in the plasma TNF-α (ΔTNF-α) concentration after LPS injection. The graph shows the different growth rates of TNF-α in different time phases within one hour after LPS stimulation. Therefore, we divided the formation rate of TNF-α ($k_{0}$, pg/(mL·h)) into two stages with a piecewise function, which provided a feasible method to describe TNF-α generation.

$$k_{0}=\left\{ \begin{aligned} 358.25\left( 0\leq t<0.33 h \right) \\ \\ \\ \\ 5200.1\left( 0.33h\leq t<1 h \right) \end{aligned} \right.$$


Figure S2. The lung LPS concentration after LPS administration via the tail vein. Male Sprague–Dawley (SD) rats (n=18) were divided randomly into 6 groups. Each group received 100 μg/kg of LPS via the tail vein (time=0). After 15, 30, 60, 90 and 120 min, the rats were anaesthetized with urethane. The lungs were collected immediately, frozen in liquid nitrogen and kept at minus eighty degrees centigrade. The lung tissues were homogenized with RIPA including PMSF using a tissue homogenizer. After high-speed centrifugation (13000 r/min, 10 min), the clear upper fluid was collected and used for detection of the LPS concentration with an ELISA kit (Shanghai ExCell Biology, Inc., Shanghai, China). The result showed no significant changes in the lung LPS concentrations after LPS (100 μg/kg) injection. Most of the endotoxin that entered the blood was combined with blood macrophages. Endotoxin injection had no effect on the lung endotoxin concentration.


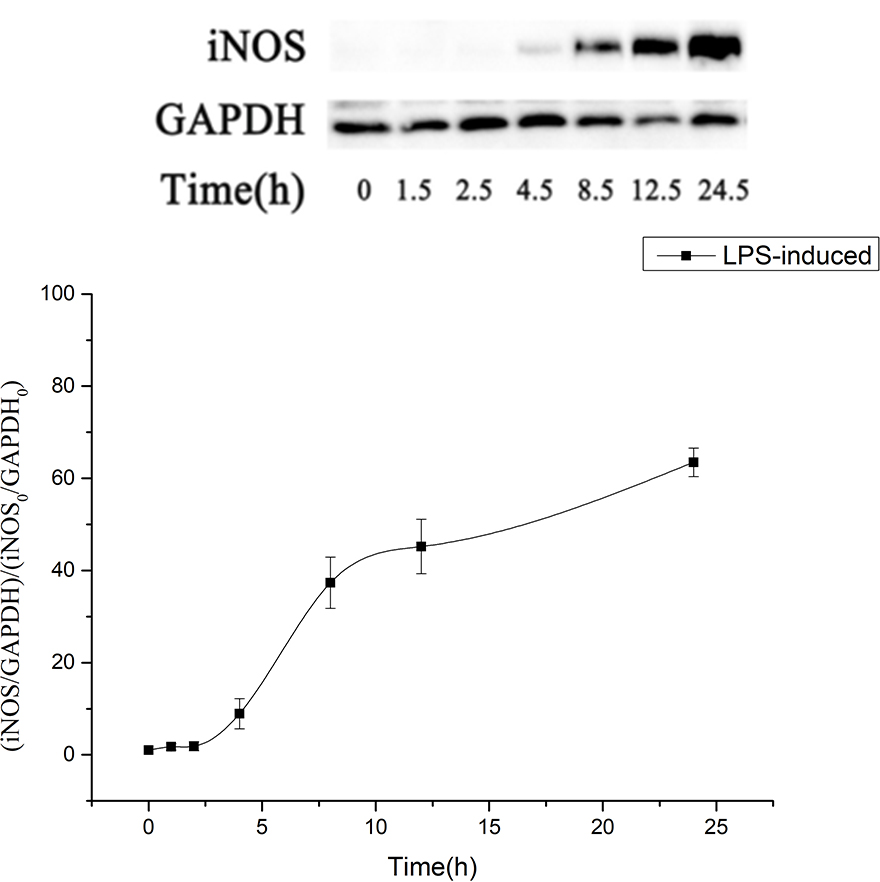


Figure S3. Time course of iNOS expression after LPS stimulation in RAW264.7 cells. RAW264.7 cells were treated with 1 μg·mL^-1^ of LPS. After 0, 1.5, 2.5, 4.5, 8.5, 12.5 and 24.5 h, the cells were washed with PBS and lysed in RIPA buffer. The cell lysates were immunoblotted with an anti-iNOS antibody. GAPDH staining is shown as a loading control. Peak iNOS expression was detected at 8.5 h.


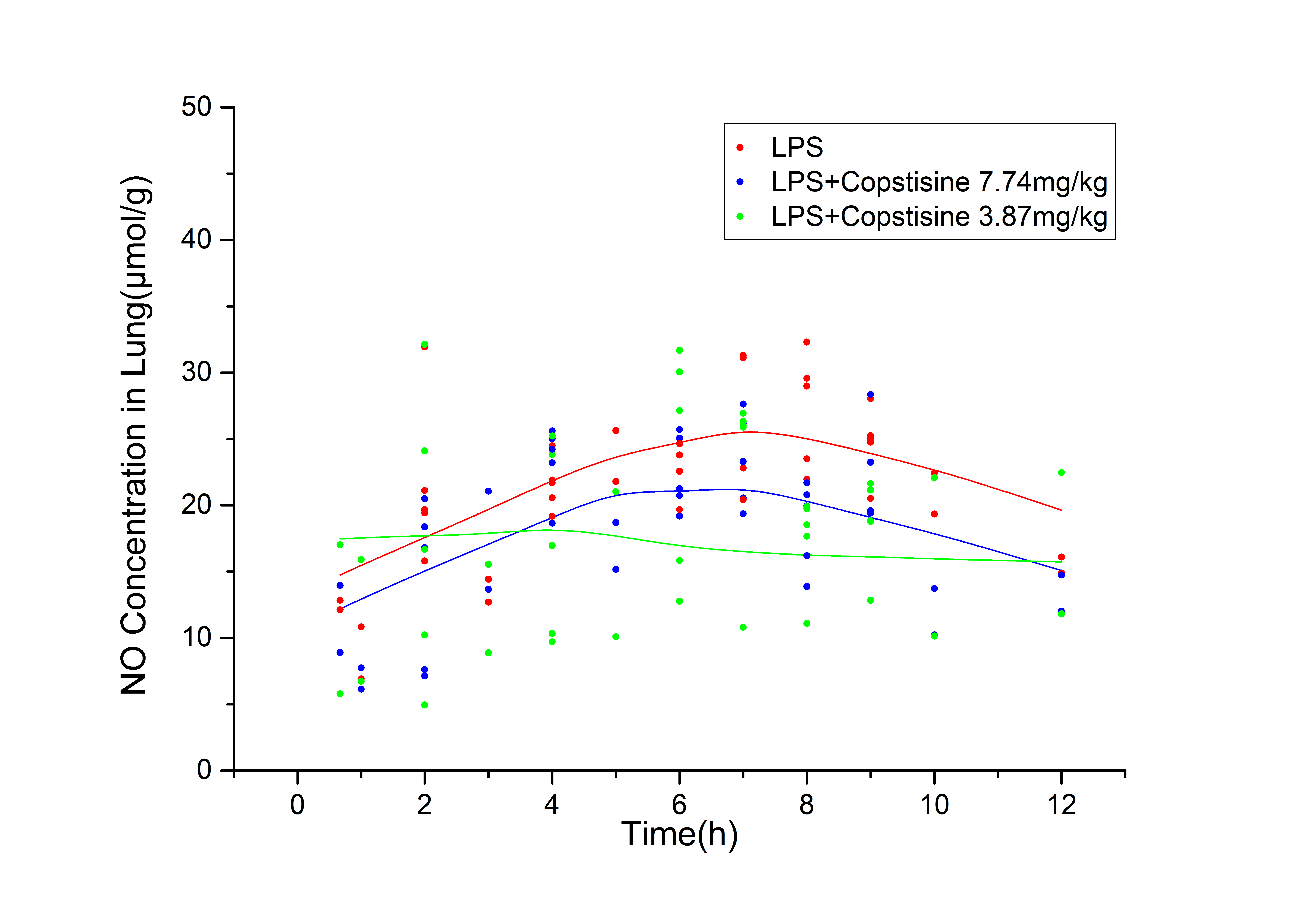


Figure S4. Time course of the lung NO concentration at various time points after LPS administration in rats. It is described as a scatter plot with its locally weighted scatterplot smoothing (LOWESS) line. Colors correspond to the LPS (●), LPS + coptisine 7.74 mg/kg (●), and LPS + coptisine 3.87 mg/kg (●) groups. The jitter procedure was used to show complete individual data. In these groups, the NO concentration in the lung was not elevated by endotoxin. Since no fluctuation in NO production was observed in lung but a peak in the plasma concentration was found at 8 h after endotoxin injection, we inferred that iNOS diffused into the blood from the lung.


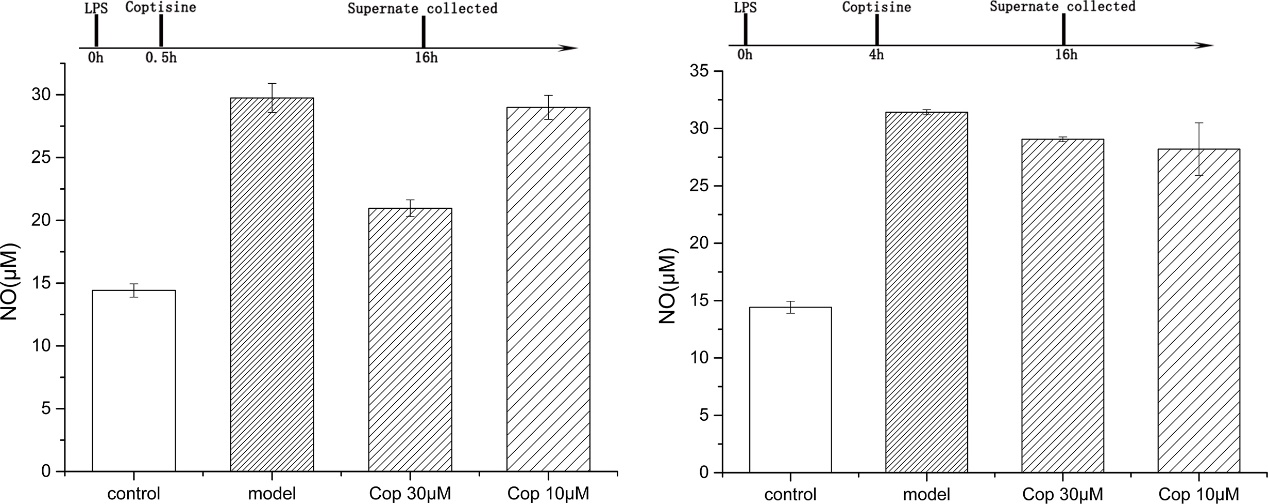


Figure S5. Effect of coptisine on the NO concentration after 0.5 and 4 h of LPS stimulation. Raw264.7 macrophage cells were induced with LPS for 0.5 h and 4 h and then incubated with coptisine (30 μM and 10 μM). After 16 h, the NO concentration in the cell supernatant was measured using a Griess kit (Beyotime Biotechnology). The data are reported are the mean values from triplicate analyses.

The results showed that the inhibitory effect of coptisine (30 μM) after 0.5 h of LPS stimulation was stronger than that after 4 h of LPS stimulation. Coptisine had few effects after TNF-α release. We inferred that the rapid elevation in the TNF-α concentration was induced early after LPS stimulation and that coptisine could inhibit the elevation at 0.5 h. In contrast, little inhibition was observed 4 h following coptisine treatment. These results indicate that TNF-α is a mediator of NO synthesis and that the major effect of coptisine is TNF-α inhibition.


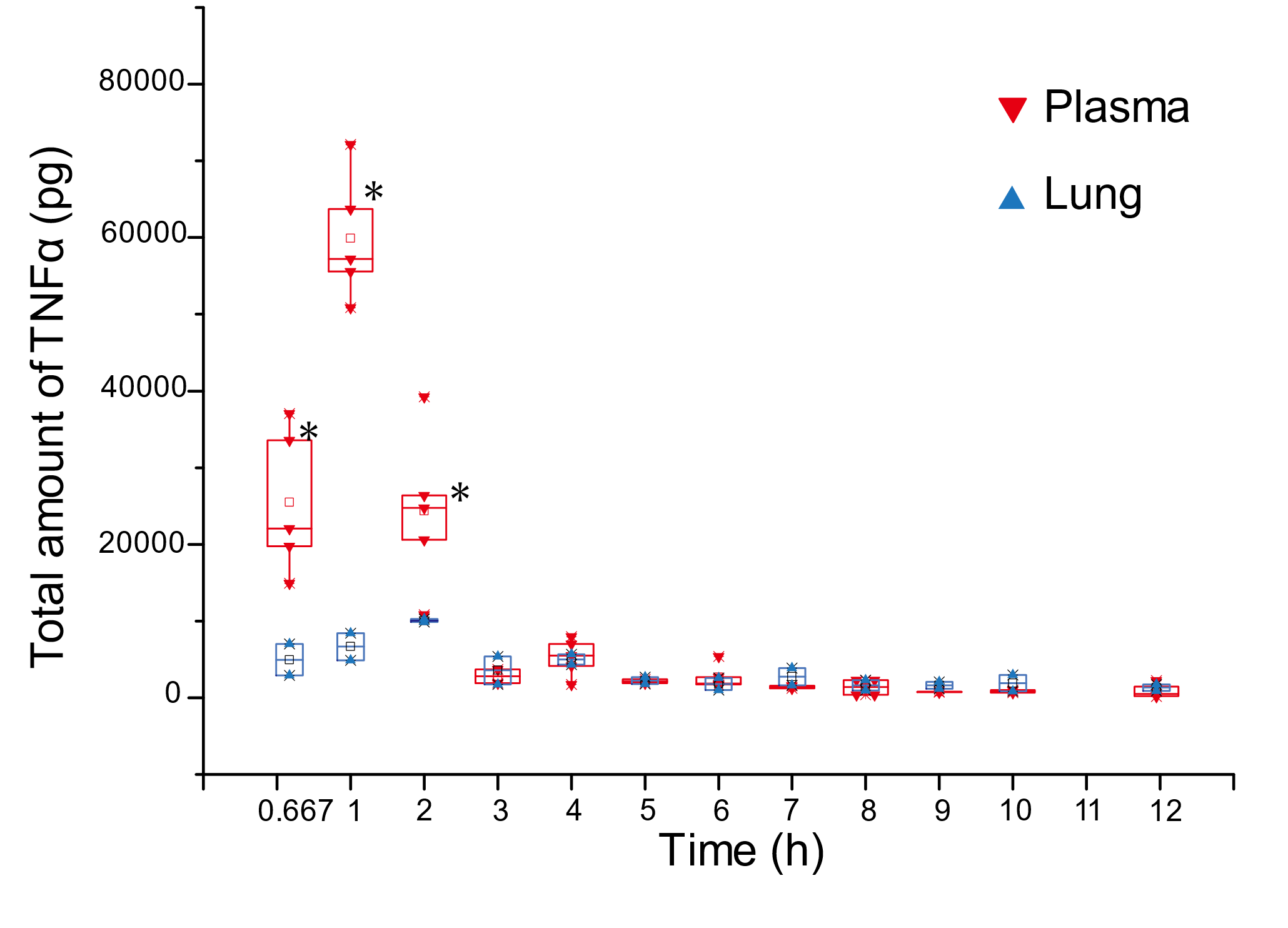


Figure S6. Total amount of the TNF-α in the plasma and lung in LPS-stimulated rats. The aim of this experiment is to compare the total amount of TNF-α in the lungs and blood. To reduce the pain of the animals, two rats were sacrificed for detection of TNF-α in the lungs at each time point, and blood from 2~5 rats was used to examine the plasma TNF-α concentration. The measured values are represented by triangles with different colors in the graph. A box plot was used to indicate the degree of dispersion. * indicates that the difference is significant in the T-test (p<0.05). When comparing the amounts of plasma and lung TNF-α, we found that TNF-α was mainly produced in the blood 1 h after endotoxin stimulation. At the 2 h time point, the plasma TNF-α was rapidly reduced, whereas the lung TNF-α continued to increase. Therefore, we can deduce that TNF-α in the lung is transferred from the blood rather than produced in the lung.


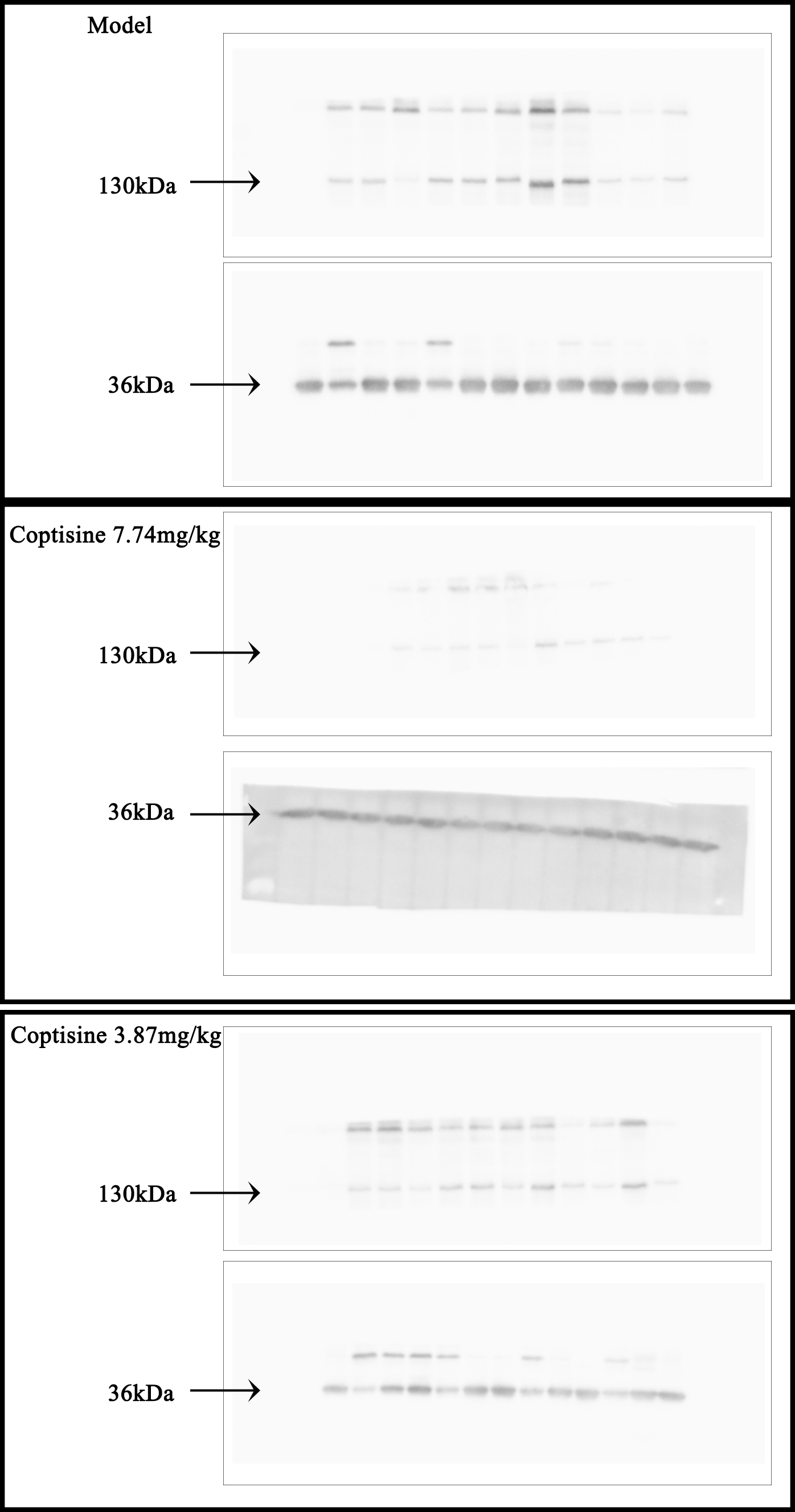


Figure S7. Full-length iNOS (130 kDa) and GADPH (36 kDa) bands.
